# Biodegradable Hydrogels for Pb^2+^ Removal from Water: Design Strategies, Mechanisms, and Future Perspectives

**DOI:** 10.3390/gels12080667

**Published:** 2026-07-25

**Authors:** Jianhui Guo, Yue Hu, Chang Ma, Wei Zhang, Youming Dong, Yida Niu, Sipei Liu, Yi Zhang, Cheng Li

**Affiliations:** 1 Department of Environmental Design, College of Fine Arts, Henan University, Kaifeng 475001, China; 13663022485@163.com (Y.H.); machang2016@163.com (C.M.); zwjan06@163.com (W.Z.); l13849148901@163.com (S.L.); 2 College of Materials Science and Technology, Nanjing Forestry University, Nanjing 210037, China; youming.dong@njfu.edu.cn; 3 College of Art, Zhengzhou University of Science and Technology, Zhengzhou 450064, China; niuyida@zit.edu.cn; 4 Faculty of Humanities and Arts, Macau University of Science and Technology, Macau 999078, China; 5 College of Forestry, Henan Agricultural University, Zhengzhou 450046, China

**Keywords:** hydrogel, cellulose, lignin, chitosan, sodium alginate, starch, lead (Pb^2+^) ions

## Abstract

Lead (Pb^2+^) pollution poses a severe threat to the ecological environment and human health due to its high toxicity, bioaccumulation, and refractory nature. Traditional treatment technologies for lead-contaminated wastewater, such as chemical precipitation, ion exchange, and membrane separation, often face limitations, including secondary pollution, high costs, and high energy consumption. In contrast, adsorption has emerged as a promising alternative technology with advantages such as a simple process, high efficiency at low concentrations, and renewability. Biomass-based hydrogels and their composite systems, as novel green adsorbent materials, combine the abundant functional groups of natural biomass with the structural stability, high porosity, and recoverability of hydrogels through a three-dimensional cross-linked network, offering unique advantages for lead ion adsorption. Depending on their composition, these systems range from fully biodegradable pure biopolymer networks to partly biodegradable or biomass-containing composites incorporating inorganic, carbon-based, or metal–organic framework (MOF) materials. This paper systematically reviews the latest research progress on cellulose, lignin, sodium alginate, chitosan, starch-based hydrogels, and their composite systems for lead (Pb^2+^) adsorption. First, the structural characteristics, cross-linking mechanisms, and functional modification strategies of various biomass hydrogels are introduced. Then, the adsorption mechanisms of Pb^2+^, including multiple modes of action such as coordination complexation, ion exchange, electrostatic interaction, and physical adsorption, are systematically analyzed. The adsorption performance of different material systems is compared in detail. The regeneration and recycling performance, as well as the potential practical applications, of the materials are evaluated. On this basis, the main challenges in current research are summarised: balancing adsorption capacity and mechanical strength, achieving selective adsorption in actual wastewater, improving regeneration efficiency, and optimizing costs. In addition, future development directions for biomass hydrogel adsorbent materials are discussed, including the design of multi-functional composite materials, the development of intelligent, responsive hydrogels, engineering-scale-up, and life-cycle assessment. This review aims to provide a theoretical framework and technical roadmap for the rational design of high-performance, sustainable hydrogel adsorbents and to promote their engineering application for the treatment of lead-contaminated wastewater.

## 1. Introduction

As industrialization accelerates, heavy metal pollution has emerged as a severe global environmental hazard. Lead ions (Pb^2+^) are non-biodegradable and prone to bioaccumulation along food chains, causing irreversible neurological damage in children and cardiovascular diseases in adults even at low exposure levels [1,2,3]. The World Health Organization has listed lead as a chemical of major public health concern [4,5,6,7]. Conventional lead wastewater treatment technologies suffer from obvious drawbacks, such as secondary sludge pollution and high operating costs [8,9,10]. By contrast, adsorption represents a low-cost and flexible alternative for Pb^2+^ removal; however, many existing biomass adsorbents still lack systematic structural optimization and mechanistic comparison [11,12,13,14].

The development of adsorbents has evolved from traditional inorganic materials, such as activated carbon and zeolites, to functionalized synthetic polymers and nanomaterials [15,16,17,18]. In this context, biomass-based hydrogels have attracted increasing attention as green and sustainable adsorbents because they are renewable, biodegradable, and rich in functional groups. Representative biomass hydrogel platforms include cellulose, chitosan, lignin, starch, and sodium alginate, which are consistent with the principles of sustainable development and green chemistry [19,20,21,22]. It is also necessary to clarify the scope of “biodegradability” in advanced biomass-based hydrogel adsorbents. Pristine biomass hydrogels composed of natural polymer networks are generally biodegradable. However, to enhance adsorption capacity, mechanical strength, separation efficiency, or long-term stability, many composite systems incorporate carbon nanomaterials, such as graphene oxide and reduced graphene oxide, magnetic particles such as Fe_3_O_4_, metal–organic frameworks, conductive polymers, or inorganic fillers. Consequently, the materials discussed in this review cover a broad spectrum, including fully biodegradable hydrogels, partially biodegradable composite hydrogels, and biomass-containing hybrid adsorbents. This distinction is important for accurately evaluating their environmental sustainability and practical application potential.

Nevertheless, raw biomass materials face several bottlenecks in engineering applications, including insufficient mechanical strength, a tendency to disperse in aqueous systems, difficult recovery, and low utilization efficiency of active sites [23,24,25,26]. Hydrogel technology addresses these issues by forming a three-dimensional cross-linked network that retains hydrophilicity while providing structural stability. Its highly porous internal structure facilitates the rapid diffusion and mass transfer of Pb^2+^, and the cross-linked network provides an ideal carrier for introducing and stabilizing active functional groups [27,28]. Despite rapid progress in this field, much of the existing literature has focused on single-material systems or individual modification strategies, while systematic comparisons among different biomass hydrogel platforms and in-depth analyses of their adsorption mechanisms remain limited. Therefore, this review addresses the following core question: how can high-efficiency, low-cost, and environmentally sustainable biomass-based hydrogel adsorbents be rationally designed for stable Pb^2+^ removal from practical lead-contaminated wastewater, and how can their underlying structure performance relationships be systematically understood.

To address this gap, this review provides a systematic and comparative overview of recent advances in Pb^2+^ adsorption by five representative biomass-based hydrogels, namely cellulose, chitosan, lignin, starch, and sodium alginate. Their preparation methods, functional modification strategies, and Pb^2+^ adsorption mechanisms, including coordination complexation, ion exchange, and electrostatic attraction, are comprehensively compared and discussed. In addition, the key factors governing adsorption performance, such as solution pH, initial Pb^2+^ concentration, competing ions, contact time, and adsorbent regeneration, are systematically summarized. Finally, this review outlines emerging directions in material design and discusses the technical challenges that must be overcome for engineering applications. Compared with previous reviews, this work aims to establish a unified structure performance framework and a rational design roadmap for high-performance, biosustainable hydrogel adsorbents, thereby bridging fundamental understanding and practical application in the remediation of lead-contaminated wastewater.

## 2. Data Collection and Methodology

To ensure a comprehensive and rigorous review of the current advancements, a systematic literature search strategy was employed. The primary databases utilized for literature retrieval were Web of Science and Google Scholar. The search was conducted using a combination of exact keywords, including “biomass hydrogel,” “lead adsorption,” “Pb^2+^,” “wastewater treatment,” and specific material identifiers (“cellulose,” “chitosan,” “lignin,” “starch,” and “alginate”). To ensure the timeliness and relevance of this review, the literature collection focused primarily on publications from the last five years. During the screening process, priority was given to studies that provided in-depth mechanistic insights into adsorption, as well as those that evaluated the regeneration capability of the hydrogels and their practical performance in real wastewater applications. Date: 30 April 2026.

## 3. Synthesis of Biomass Composite Hydrogels

### 3.1. Structural Characteristics and Functional Advantages of Biomass Composite Hydrogels

**Figure 1:**
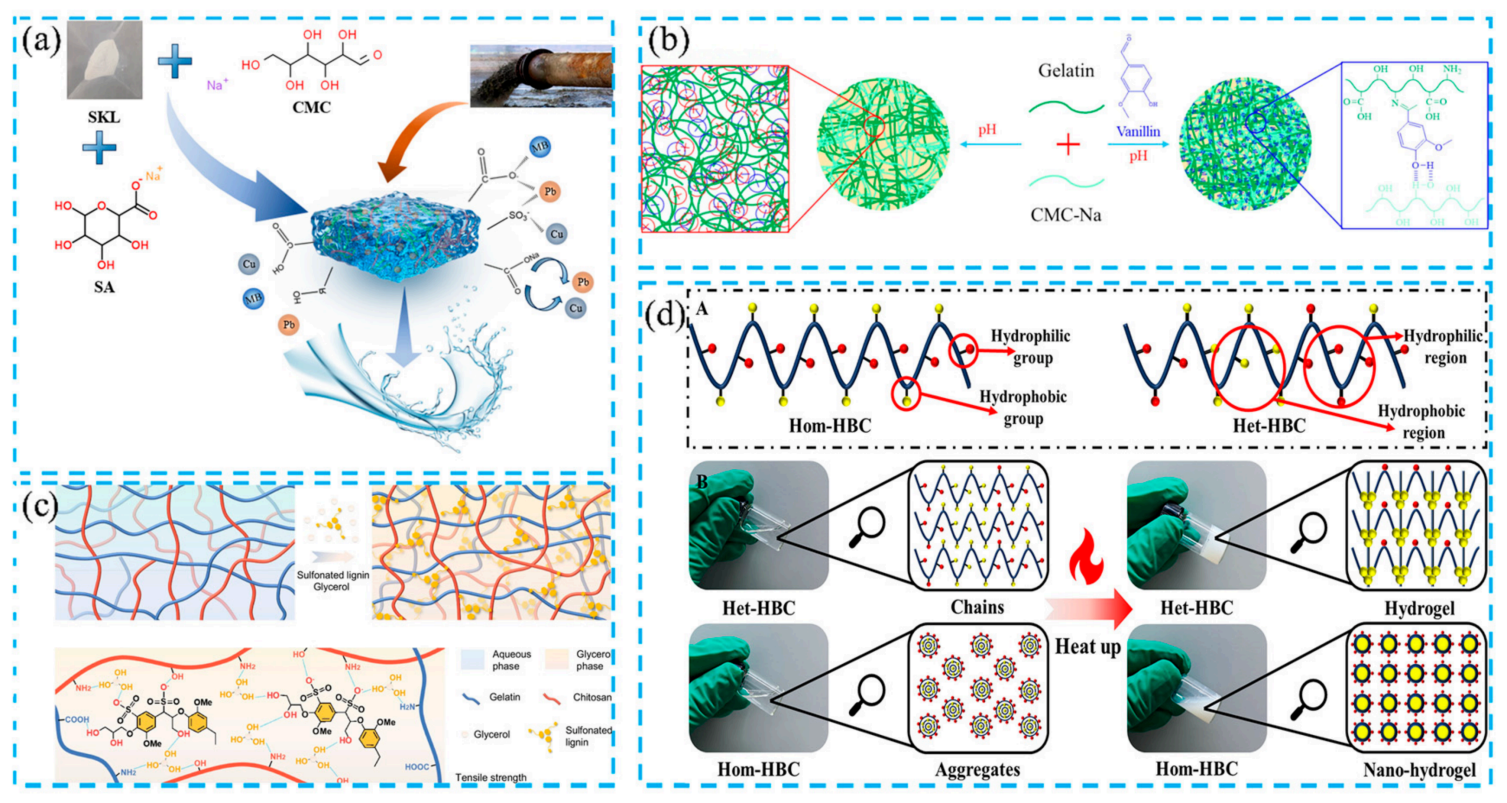
(**a**) Preparation of SA/CMC/SKL hydrogel adsorbent and its adsorption properties for heavy metals and dyes in wastewater. Reproduced with permission from reference [30]. Copyright 2025, Elsevier. (**b**) Schematic mechanism diagram of the gel-coacervation system of coumarin-modified gelatin-CMC-Na complex. Reproduced with permission from reference [34]. Copyright 2025, Elsevier. (**c**) Schematic illustration of the preparation of CGSD biogel and the mechanism of synergistic enhancement of non-covalent bonds within the CGSD biogel. Reproduced with permission from reference [35]. Copyright 2025, Springer Nature. (**d**) Assembly mechanism of Het-HBC and Hom-HBC. Reproduced with permission from reference [36]. Copyright 2025, Elsevier.

Hydrogels, as polymeric materials with three-dimensional cross-linked networks, can absorb large amounts of water in aqueous media while maintaining structural integrity. Their inherent hydrophilicity and high water-retention capacity endow them with great potential for environmental remediation, particularly for the capture of heavy metal ions [29]. Biomass composite hydrogels are hybrid material systems formed by molecular-level compounding of natural biomass components (such as cellulose, chitosan, lignin, and alginate) with hydrogel matrices (Figure 1a) [30,31]. Such materials not only retain the renewability and biodegradability of biomass but also achieve synergistic improvements in mechanical properties, structural stability, and functional diversity by forming cross-linked networks, thereby significantly enhancing their practical performance in controlling heavy metal pollution.

The advantages of biomass composite hydrogels are reflected in their efficient adsorption performance. The three-dimensional porous network structure provides abundant active adsorption sites, offering excellent capture capacity for Pb^2+^ and adsorption capacities several times higher than those of traditional materials [32]. Meanwhile, the raw materials are derived from renewable biomass and are biodegradable, aligning with the principles of green chemistry and sustainable development and effectively reducing the risk of secondary pollution [33]. In addition, strategies such as regulating cross-linking density, introducing functional groups, or incorporating nano-reinforcement phases can precisely adjust the pore structure, surface chemical properties, and responsive behavior, thereby enabling on-demand customization of material properties. Compared with single-component hydrogels, the biomass composite system significantly improves mechanical strength and anti-fatigue performance through synergistic reinforcement, ensuring the material maintains structural integrity during long-term operation. The integration of the above characteristics makes biomass composite hydrogels a new generation of lead ion adsorption materials.

### 3.2. Preparation Method of Biomass Composite Hydrogels

The preparation methods for biomass composite hydrogels include physical cross-linking, chemical cross-linking, copolymerization, and self-assembly. By regulating network topology and interfacial chemical properties, these different construction strategies can precisely optimize lead ion adsorption performance.

The chemical cross-linking method covalently links biomass components to polymer matrices, forming a stable three-dimensional network skeleton. Meng et al. [34] utilized the Schiff base reaction between coumarin aldehyde groups and gelatin amino groups to form covalent cross-links, while the hydroxyl groups of coumarin also constructed a hydrogen-bonding network with CMC-Na and gelatin. This synergistic effect of covalent/non-covalent dual cross-linking enhances the binding strength between wall materials and significantly improves the mechanical properties of the gel. Common cross-linking agents include glutaraldehyde, epoxy compounds, and multivalent metal ions (e.g., Ca^2+^). Among these, the regulation of cross-linking density directly influences porosity, specific surface area, and pore-size distribution, thereby determining the accessibility of active sites and the efficiency of ion transport (Figure 1b).

The copolymerization strategy involves chain-growth polymerization of biomass-derived and vinyl monomers, such as acrylic acids and acrylamides, via radical or ionic polymerization. This process not only introduces functional groups into the molecular chain segments but also synergistically enhances mechanical properties and adsorption capacity. On this basis, Gu et al. [35] introduced lignin into the chitosan/gelatin double network and replaced the solvent with glycerol via the “lignin/solvent synergistic induced non-covalent enhancement” strategy, thereby enabling precise regulation of the network topology. This method has enabled the material to overcome the mechanical property limitations of traditional double-network biogels, achieving tensile strengths of 4.35 ± 0.08 MPa and compressive strengths of 66.11 ± 3.90 MPa. Solution cross-linking technology is based on the dissolution-regeneration process of biomass. By controlling parameters such as the concentration of cross-linking agents, reaction temperature, and solvent evaporation rate, the microscopic morphology and surface charge distribution of the gel can be precisely adjusted. This method is easy to operate and highly reproducible (Figure 1c).

Physical self-assembly relies on non-covalent forces, such as hydrogen bonding, electrostatic interactions, and hydrophobic interactions, to drive the ordered arrangement of components, forming a dynamic, reversible network. This approach avoids the use of organic cross-linking agents, aligns with the concept of green preparation, and simultaneously endows the material with environmental responsiveness and self-healing capability. Bi et al. [36] discovered that the structural differences between heterogeneously modified HBC (Het-HBC) and homogeneously modified HBC (Hom-HBC) originate from the alkyl distribution pattern. In homogeneous modification, alkyl groups are uniformly grafted onto the hydroxyl and amino groups of chitosan, inducing intramolecular self-assembly to form nanoparticles. In contrast, in heterogeneous modification, alkyl groups exhibit localized aggregation or a sparse distribution, thereby promoting intermolecular assembly to form a porous hydrogel network (Figure 1d).

## 4. Adsorption Mechanism of Pb^2+^ by Biomass Hydrogels

The process of lead ion capture by biomass hydrogels involves the synergistic action of physical and chemical adsorption. The adsorption efficiency of hydrogels depends primarily on the types and densities of functional groups on the material surface and on the strength of the interactions between these groups and the target ions. An in-depth analysis of the adsorption mechanism is highly informative for the rational design of high-performance materials.

### 4.1. Mechanism of Physical Adsorption

**Figure 2:**
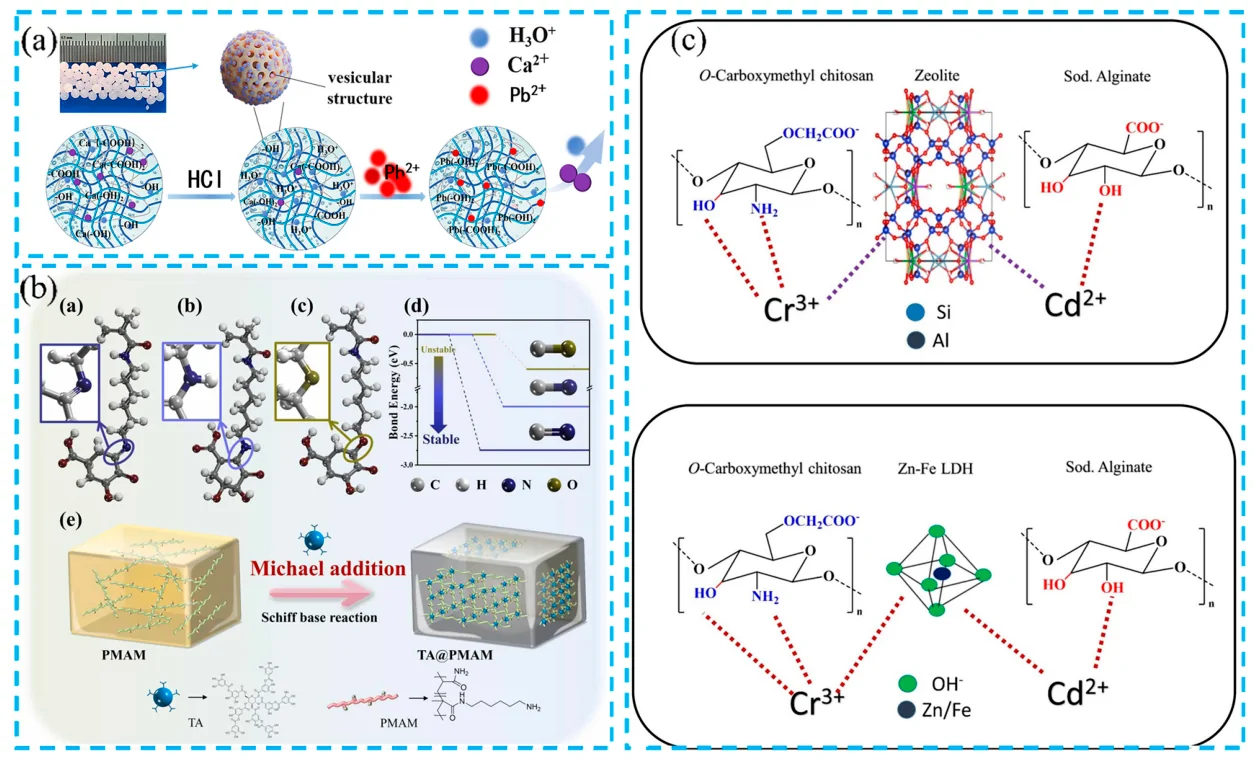
(**a**) Protonated 3D network gels for highly efficient capture of Pb^2+^ from contaminated wastewater. Reproduced with permission from reference [37]. Copyright 2025, Elsevier. (**b**) Cartoon illustration of tannic acid-based covalent polymeric hydrogel adsorbent (TA@PMAM). Reproduced with permission from reference [38]. Copyright 2024, Elsevier. (**c**) Proposed heavy metal interaction mechanism between ACZ and (**b**) ACL. Reproduced with permission from reference [39]. Copyright 2024, Elsevier.

Physical adsorption primarily relies on non-covalent intermolecular forces to accumulate Pb^2+^ at the surface of hydrogels, including hydrogen bonding, van der Waals forces, and electrostatic attraction. The abundant oxygen-containing functional groups, including hydroxyl (-OH) and phenolic hydroxyl groups, within the three-dimensional network of hydrogels can act as either hydrogen bond acceptors or donors, forming hydrogen bond bridges with hydrated Pb^2+^. This interaction significantly contributes to the initial adsorption rate in low-concentration pollution systems. Although van der Waals forces are relatively weak at individual interaction sites, they can exert a cumulative effect through the high specific surface area provided by the porous structure, thereby promoting the retention of Pb^2+^ near organic groups such as alcohols and phenols. Electrostatic attraction arises from the Coulombic interactions between negatively charged groups (e.g., carboxyl-COO^−^ and sulfate-OSO_3_^−^) on the surface of biomass components and Pb^2+^. As shown in Figure 2a, this mechanism is particularly prominent in alginate-based hydrogels, where the adsorption strength is significantly regulated by solution pH and ionic strength [37]. The physical adsorption process is typically reversible; when environmental conditions (temperature, pH, and ion concentration) change, the already adsorbed Pb^2+^ may desorb. This characteristic not only enables material regeneration but also poses challenges to stability in practical applications.

### 4.2. Mechanism of Chemical Adsorption

Chemical adsorption provides strong binding of Pb^2+^ to the hydrogel matrix mainly via covalent or coordinate bonds with specific functional groups, which are much stronger than those in physisorption and usually irreversible [38]. In lignin-based systems, functional groups such as phenolic hydroxyl (-OH), carboxyl (-COOH), and sulfonate (-SO_3_^−^) play critical roles in Pb^2+^ binding. Ion exchange is an important chemical adsorption pathway: protonated active sites, such as amino groups (-NH_3_^+^) and carboxyl groups (-COOH), can undergo ion-exchange reactions with Pb^2+^ in solution. For instance, amino groups in chitosan hydrogels capture Pb^2+^ by releasing counterions (H^+^, Na^+^), forming stable Pb-hydrogel complexes [40]. Coordination involves the formation of coordinate bonds between Pb^2+^ (acting as Lewis acids) and electron-donating groups, including amino groups, phenolic hydroxyl groups, and carboxyl groups. The nitrogen atoms with lone-pair electrons on chitosan chains and the phenolic hydroxyl groups in lignin serve as coordination centers, forming thermodynamically stable chelates through multidentate coordination, thereby enhancing adsorption selectivity and irreversibility [41]. It is important to emphasize that Pb^2+^ binding is governed by complexation, ion exchange, and electrostatic attraction rather than hydrogen bonding or π interactions, since Pb^2+^ is a metal ion, not an aromatic molecule. Due to strong chemical adsorption, Pb^2+^ is difficult to desorb using simple physical methods, favoring long-term immobilization but increasing the challenges of adsorbent regeneration.

### 4.3. Regulatory Effects of Surface Functional Groups

The chemical properties and spatial distribution of surface functional groups essentially determine the adsorption performance of biomass hydrogels. As the most common hydrophilic group, hydroxyl groups enhance the hydration capacity of materials through hydrogen-bonding networks and provide primary adsorption sites, which play a key role in adsorption kinetics under low-pollution load conditions. Amino groups have both alkalinity and nucleophilicity: protonated -NH_3_^+^ can rapidly capture Pb^2+^ through electrostatic interaction, while unprotonated lone pair electrons participate in a coordination reaction. This dual functionality confers pH-dependent adsorption behavior on chitosan-based materials [39]. Due to their strong acidity and high charge density, carboxyl groups exist in deprotonated form (-COO^−^) under neutral to alkaline conditions and capture Pb^2+^ [42]. The density, accessibility, and chemical activity of functional groups together constitute the microscopic basis of adsorption performance. Introducing high-affinity groups, such as thiol (-SH) and phosphonic acid (-PO_3_H_2_), via chemical modification, or optimizing their spatial arrangement via grafting, cross-linking, and other methods, has become a core strategy for improving the lead-ion-removal capacity of materials.

## 5. Lead Ion Adsorption of Biomass Composite Hydrogels

### 5.1. Cellulose-Based Hydrogels for Lead Ion Adsorption

Cellulose, the most abundant renewable polysaccharide in nature, is composed of β-D-glucose units linked by β-1,4-glycosidic bonds. Its molecular chains are rich in hydroxyl groups, endowing it with excellent hydrophilicity and biodegradability, making it an ideal substrate for the construction of environmentally friendly adsorptive hydrogels. However, the cellulose skeleton contains only hydroxyl groups as functional groups, resulting in low active site density and insufficient adsorption capacity and selectivity for Pb^2+^. To overcome this limitation, current modification strategies mainly include physical doping, chemical grafting, and multi-component composites. By introducing high-affinity functional groups (such as carboxyl, amino, and sulfhydryl groups) or by constructing specific microstructures, the densification and functionalization of adsorption sites are achieved, thereby improving the material’s capture efficiency for heavy metal ions.

**Figure 3:**
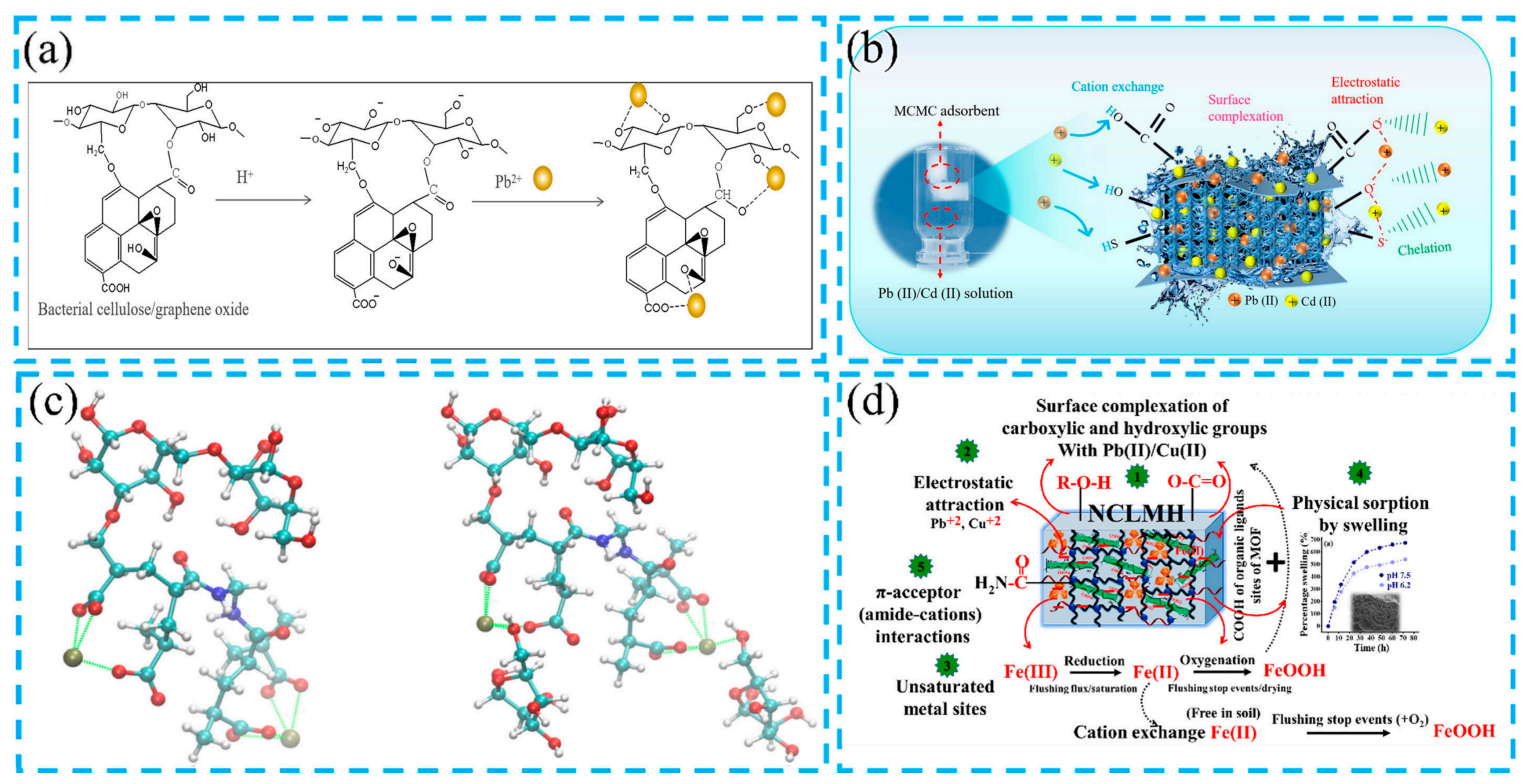
(**a**) Adsorption mechanism of Pb^2+^ by BC/GO-1. Reproduced with permission from reference [43]. Copyright 2024, MDPI. (**b**) Schematic diagram of the adsorption mechanism of Pb^2+^ and Cd (II) by MCMC adsorbent [46]. Copyright 2025, Elsevier. (**c**) Each PAAc unit binds one Pb^2+^ ion via two carboxylate ions. Reproduced with permission from reference [47]. Copyright 2023, Springer Nature. (**d**) Removal mechanism of Pb and Cu induced by NCLMH adsorbent. Reproduced with permission from reference [50]. Copyright 2024, Elsevier.

To address the limited binding sites for cellulose, researchers have enhanced chemiosorption via oxygen-containing groups by introducing carbon materials and regulating their structures. Absorption processes in many of the materials studied conform to adsorption isotherm models such as the Langmuir or Freundlich models and often follow pseudo-first- or pseudo-second-order kinetic models. The Langmuir isotherm assumes monolayer adsorption onto a finite number of uniform sites and is often used to quantify maximum adsorption capacity (Qmax). The Freundlich isotherm, on the other hand, describes adsorption on heterogeneous surfaces and the formation of multilayers. In terms of kinetics, the pseudo-first-order model typically describes physical adsorption, where the rate depends on the number of unoccupied sites, whereas the pseudo-second-order model indicates chemisorption involving electron sharing or transfer, suggesting stronger binding interactions between the adsorbate and the adsorbent. Zhang et al. [43] synthesized a three-dimensional porous bacterial cellulose/graphene oxide (BC/GO) composite hydrogel using an ultrasonic oscillation process (as shown in Figure 3a). Structural and surface features were thoroughly characterized by TEM, SEM, FTIR, NMR, and Zeta potential analyses, confirming the successful disruption of intra- and intermolecular hydrogen bonds in bacterial cellulose, thus exposing more hydroxyl (-OH) groups. The introduced carboxyl (-COOH) groups on graphene oxide synergistically enhanced electrostatic attraction toward Pb^2+^ ions. The adsorption process was well described by the Freundlich isotherm model, indicative of heterogeneous surface sites and multilayer adsorption.

Meanwhile, the adsorption kinetics conformed to the pseudo-second-order model, suggesting that chemiosorption via electron sharing or electron transfer dominated the binding mechanism (Table 1). The BC/GO hydrogel demonstrated a maximum adsorption capacity of 224.5 mg/g for Pb^2+^, illustrating the effectiveness of oxygen-containing groups in enhancing heavy metal uptake. To further enhance selectivity and adsorption binding energy, Pan et al. [44] used a chemical modification strategy to prepare freeze-dried FCMC/FGO (amino acid-functionalized carboxymethyl cellulose/amino acid-functionalized graphene oxide) aerogels via grafting, mixing, and cross-linking. SEM, EDS, FTIR, and XPS characterizations revealed well-developed porous networks and abundant functional groups facilitating adsorption. The aerogel’s Pb^2+^ adsorption followed a Langmuir isotherm, indicating monolayer adsorption on uniform sites, and the kinetics were described by a pseudo-second-order model, reinforcing chemiosorption as the main interaction. The maximum capacity was significantly higher at 304.6 mg/g. Notably, after six adsorption–desorption cycles, the material retained high stability with only a 14% decrease in Pb^2+^ removal efficiency, demonstrating excellent reusability.

To further enhance selectivity and adsorption binding energy, chemical modification strategies have been employed to precisely regulate molecular orbital interactions. Badawy et al. [45] synthesized a cross-linked hydroxyethyl cellulose/acrylic acid hydrogel via gamma-ray irradiation, which was further modified with surfactants. FTIR, SEM, and EDX analyses confirmed successful functionalization. Their adsorption data fit the Freundlich isotherm, indicating adsorption on heterogeneous surfaces, but the kinetics fit a pseudo-first-order model, suggesting the predominance of physical adsorption or weaker chemisorption. Thermodynamic analyses showed the process was spontaneous and endothermic, with positive entropy changes, suggesting increased disorder at the solid–liquid interface. Surfactants enhanced electrostatic attraction and possibly ion-pair formation, although maximum capacity remained moderate (26.65 mg/g). To overcome the limitation of surfactant modification in adsorption capacity, Pan et al. [46] prepared a modified carboxymethyl cellulose (CMC)-based adsorbent with a three-dimensional network structure by cross-linking CMC with 3-mercaptopropyltrimethoxysilane, followed by freeze-drying. FTIR, XPS, and density functional theory (DFT) calculations supported the formation of strong chemical bonds between Pb^2+^ and thiol groups. This adsorbent exhibited a maximum adsorption capacity of 322.96 mg/g for Pb^2+^, achieving adsorption equilibrium within 60 min. After five adsorption–desorption cycles, the adsorbent maintained a high adsorption efficiency. The adsorption process conformed to the pseudo-second-order kinetic model and the Langmuir adsorption isotherm, suggesting that the adsorption was predominantly chemical. Density functional theory (DFT) calculations revealed a significant reduction in the HOMO-LUMO energy gap of the system upon adsorption, suggesting orbital hybridization between Pb^2+^ and functional groups, such as the thiol groups introduced by modification, thereby enhancing charge-transfer ability. This mechanism combined multiple interactions, including electrostatic attraction, surface complexation, chelation, and ion exchange (Ca^2+^/Na^+^ ↔ Pb^2+^), enabling efficient capture of Pb^2+^ (as shown in Figure 3b).

In studies pursuing ultra-high adsorption capacities (>900 mg/g), the synergistic effect of multi-component biomass primarily manifests as enhanced binding energy and multiple active sites (Table 1). Fajardo et al. [47] incorporated cellulose nanocrystals (CNWs) into starch hydrogels grafted with polyacrylate (PTA) to prepare aqueous Pb^2+^ removal materials. Only 25 mg of the composite hydrogel, with a contact time of 60 min, was required to achieve a high adsorption capacity of 935.8 mg/g. FTIR and DFT studies showed that hydroxyl groups on CNWs contributed an additional 30 kcal/mol to the binding energy of Pb^2+^, substantially promoting chemiosorption (as shown in Figure 3c). This process was described as a combination of chemiosorption and intraparticle diffusion. Similarly, to achieve ultra-high adsorption capacity, Yang et al. [48] fabricated a composite gel bead integrating carboxymethyl cellulose, sodium alginate, and zein via physical mixing and Ca^2+^ cross-linking, characterized by SEM-EDS, XRD, FTIR, and XPS. The maximum adsorption capacity of this composite gel reached 924.21 mg/g. When tested with algal concentrates, these gel beads achieved a Pb^2+^ removal rate of 92%. The adsorption mechanism involves ion exchange between the Ca^2+^ cross-linked network and Pb^2+^ during the adsorption process, while carboxyl groups (-COO^−^) and amino groups (-NH_2_) participate in coordination bonding. Additionally, co-existing substances such as glutamic acid and aspartic acid present in the system further enhance the coordination effect. This synergistic mechanism involving multiple functional groups is key to achieving monolayer chemiosorption (as described by the Langmuir model) and an extremely high adsorption capacity.

As adsorption materials move toward practical applications, mechanistic research has expanded to include the synergistic effects of environmental factors and the design of specialized structures. Fang et al. [49] reported a novel multi-functional double-layer hydrogel that integrates interfacial evaporation and adsorption functions. FTIR and SEM analyses confirmed the integrity of functional groups and the porous structure, while Zeta potential measurements revealed surface charge variations that contributed to ion adsorption. Under 1 h of solar irradiation, the evaporator reached an evaporation rate of 2.72 kg·m^−2^·h^−1^, with an evaporation efficiency of 92.91%. The introduction of solar-driven interfacial evaporation (SDIE) technology increased the adsorption capacity for Pb^2+^ from 38.67 mg/g to 83.54 mg/g. The local temperature and concentration gradients induced by interfacial evaporation accelerate ion mass transfer, thereby improving adsorption performance at both the physical and chemical levels. To meet the demand for in situ remediation of heavy metal-contaminated soil, Shaghaleh et al. [50] prepared a novel TEMPO-cellulose/lignin/acrylamide@MIL-100 (Fe) nanocomposite hydrogel adsorbent (NCLMH). Characterizations by FTIR, SEM, XRD, XPS, and Zeta potential at varying pH confirmed the presence of abundant carboxyl and hydroxyl groups, stable MOF incorporation, and a surface charge conducive to Pb^2+^ binding. The maximum theoretical adsorption capacity of NCLMH for Pb^2+^ is 416.39 mg/g at pH 6.5. The multi-pulse soil flushing mode (MF@NCLMH) achieved the highest Pb removal rate of 96.5%. The mechanism involves electrostatic attraction between carboxyl/hydroxyl groups, complexation with unsaturated Fe(III) sites in the MOF, and the gradual oxidation of Fe(II) released from the hydrogel to form FeOOH, which acts as a newly formed adsorbent to further capture heavy metals (as shown in Figure 3d). In terms of preparation process innovation, Ibebunjo et al. [51] used selective laser sintering (SLS) 3D printing technology to fabricate cellulose-polyamide composite filters via a simple method, which were then functionalized with citric acid for Pb^2+^ removal. FTIR, SEM, Zeta potential, TGA, and XPS confirmed successful functional group grafting, porous morphology, favorable surface charge, thermal stability, and chemical composition. At the optimal pH of 5.5, the maximum adsorption capacity for Pb^2+^ is 19.82 mg/g, and the adsorption equilibrium is reached within 4 h. CA/3D-MCC can be successfully recovered and regenerated over five cycles, with the removal efficiency maintained between 70% and 82%. The adsorption process is chemiosorption driven by carboxyl and hydroxyl groups. At the micro level, it still follows Langmuir monolayer adsorption and pseudo-second-order kinetics, and rapidly captures Pb^2+^ at the surface of fluid channels via electrostatic interactions and coordination binding.

**Table 1:** Summary of Cellulose biomass-based adsorptive hydrogels/aerogels/composite gels for Pb^2+^.

Biomass Base	Specific Hydrogel Material (Abbreviation)	Max Adsorption Capacity (q_max_, mg/g)	Optimal pH	Kinetic Model	Isotherm Model	Thermodynamic Insight	Ref.
Cellulose	Bacterial cellulose/graphene oxide (BC/GO)	224.5	/	Pseudo-second-order	Freundlich	/	[43]
Cellulose	Amino acid functionalized CMC/GO (FCMC/FGO)	304.6	/	Pseudo-second-order	Langmuir	/	[44]
Cellulose	Hydroxyethyl cellulose/acrylic acid + surfactants	26.65	/	Pseudo-first-order	Freundlich	Spontaneous, Endothermic	[45]
Cellulose	Silane cross-linked carboxymethyl cellulose (CMC)	322.96	/	Pseudo-second-order	Langmuir	Reduced HOMO-LUMO gap	[46]
Cellulose	Double-layer hydrogel (Solar-driven evaporator)	83.54	/	/	/	/	[49]
Cellulose	Citric acid-functionalized cellulose-polyamide (CA/3D-MCC)	19.82	5.5	Pseudo-second-order	Langmuir	/	[51]
Starch/Cellulose	Starch hydrogels grafted with polyacrylate + CNWs	935.8	/	/	/	+30 kcal/mol binding energy	[47]
Cellulose/Alginate	CMC/sodium alginate/zein composite	924.21	/	/	Langmuir	/	[48]
Cellulose/Lignin	TEMPO-cellulose/lignin/acrylamide@MIL-100(Fe) (NCLMH)	416.39	6.5	/	/	/	[50]

Note: / indicates data not specified or unavailable in original reports; BC/GO bacterial cellulose/graphene oxide; FCMC/FGO amino acid functionalized carboxymethyl cellulose/amino acid functionalized graphene oxide; CMC carboxymethyl cellulose; FCMC amino acid-functionalized CMC.GO graphene oxide; CNWs cellulose nanocrystals; PTA polyacrylate; SDIE solar-driven interfacial evaporation; MIL-100(Fe) metal–organic framework MIL-100 loaded with Fe; NCLMH TEMPO-cellulose/lignin/acrylamide@MIL-100(Fe) nanocomposite hydrogel; CA/3D-MCC citric acid functionalized 3D-printed cellulose polyamide composite filter;3D-MCC 3D-printed microstructured cellulose polyamide composite.

### 5.2. Lignin-Based Hydrogels for Lead Ion Adsorption

As an abundant natural aromatic polymer in the plant kingdom, lignin exhibits a natural coordination affinity for Pb^2+^ and excellent physicochemical stability by virtue of the dense active groups in its backbone, such as phenolic hydroxyl and methoxyl groups. To meet the growing demand for improved adsorption performance, recent studies have focused on leveraging lignin’s modifiability to construct functional hydrogels through strategies such as chemical grafting, microstructure regulation, and nanocomposite formation, aiming to optimize the pore structure and active site distribution, thereby significantly enhancing the removal efficiency of Pb^2+^ from water.

By regulating and modifying lignin’s structure through micro-morphology optimization, researchers have significantly enhanced adsorption performance (Table 2). Sun et al. [52] grafted acid hydrolyzed lignin onto a polyacrylic acid network to synthesize a pH-sensitive AHL-g-PAA composite hydrogel, achieving a Pb^2+^ adsorption capacity of 235 mg/g. The adsorption process was well described by the Langmuir isotherm and pseudo-second-order kinetic model, indicating that the mechanism is predominantly monolayer chemisorption. Building upon this, Zhang et al. [53] prepared lignocellulose gel (LCG) hydrogels with varying lignin contents via a dissolution-regeneration method. SEM, FTIR, and confocal imaging revealed that 11.6% lignin content optimally balanced structural uniformity and available active sites, resulting in a maximal Pb^2+^ adsorption capacity of 86.1 mg/g. Their findings indicated that adsorption was primarily governed by chemisorption, accompanied by multiple diffusion mechanisms. Similarly, Shen et al. [54] compared pure cellulose and lignocellulose microbeads through SEM, FTIR, and XRD analyses, finding that an appropriate amount of residual lignin promoted pore formation and increased hydroxyl groups. This structural contribution raised the Pb^2+^ adsorption capacity of lignocellulose microbeads to 0.161 mmol/g. However, excessive lignin led to particle aggregation and blockage of active sites, which should be avoided to maintain adsorption efficiency.

To overcome the limitations of single-component materials, integrating carbon-based materials and inorganic minerals into nanocomposites has become a vital strategy (Table 2). In one work, Sohni et al. [55] synthesized lignin nanoparticles (LNPs) from walnut shells, which were incorporated into a reduced graphene oxide (rGO) hydrogel network, forming rGO@LNP. SEM, FTIR, and XRD analyses confirmed successful functionalization. This composite achieved 71% Pb^2+^ removal efficiency at pH 5. The adsorption was attributed to chelation via ionized carboxyl, sulfonic acid, and hydroxyl groups on LNPs, thereby enhancing the stability of the metal-ligand complex. Moreover, Lewis acid-base interactions between graphene and Pb^2+^ further improved the removal efficiency. In another study, Li et al. [56] synthesized a lignosulfonate-modified graphene hydrogel (LS-GH) via a one-step method. SEM and FTIR analyses confirmed successful functionalization. Benefiting from a three-dimensional porous structure and abundant oxygen-containing functional groups, LS-GH showed an ultrahigh Pb^2+^ adsorption capacity of 1210 mg/g. Under dynamic conditions, it achieved 1308 mg/g within 40 min, demonstrating its practical applicability as a flexible packed-column adsorbent. From the perspective of inorganic mineral reinforcement, Ma et al. [57] developed an LBPAA (Lignin-grafted N, N′-methylene-bisacrylamide (LM) copolymerized with acrylic acid (AA))- organic montmorillonite (OrgMMT) nanocomposite, characterized by SEM and FTIR. The Pb^2+^ adsorption was pH-dependent and conformed to the Freundlich multilayer isotherm, following pseudo-second-order kinetics. The removal mechanism involved spontaneous chemical complexation, where Pb^2+^ formed Pb–O bonds with hydroxyl and carboxyl groups on the composite.

Further expanding the understanding of copolymer modification and practical use, Yao et al. [58] synthesized a bentonite/sodium lignosulfonate copolymer hydrogel (BLPA-MA) with acrylamide and maleic anhydride. SEM, FTIR, and XPS analyses, combined with kinetic and isotherm studies, showed that Pb^2+^ adsorption followed pseudo-second-order kinetics and Langmuir isotherm, indicating monolayer chemisorption. Thermodynamic parameters indicated that adsorption was spontaneous and endothermic, driven primarily by chelation involving amide, carboxyl, and sulfonic acid groups, as well as ion exchange between Na^+^ and Pb^2+^. Regarding the optimization of copolymer network structure, Munguía-Quintero et al. [59] reported a composite synthesized via conventional graft copolymerization of lignosulfonate (Ls), acrylic acid (AA), acrylamide (AM), and N, N’-methylenebisacrylamide (MBA), which has a maximum adsorption capacity for Pb^2+^ of 111.94 mg/g. SEM, FTIR, and XPS characterizations were performed. The adsorption data fitted well with pseudo-second-order kinetics and the Langmuir isotherm, indicating that adsorption capacity was closely related to the polymer network’s pore size, with a maximum Pb^2+^ capacity of 111.94 mg/g. For practical soil remediation, Li et al. [60] developed a magnetic Fe_3_O_4_@lignin hydrogel for paddy soil treatment. Characterized by SEM, EDS, FTIR, XRD, and XPS, this composite reduced Pb concentrations by approximately 25% and improved soil nutrient content. In the complex soil environment, the material’s Pb^2+^ removal was governed by multiple mechanisms, including bentonite adsorption, electrostatic attraction, ion exchange, surface precipitation, nano-effects, and complexation.

### 5.3. Sodium Alginate-Based Hydrogels for Lead Ion Adsorption

Sodium alginate, a carboxyl-rich marine natural polysaccharide, has emerged as an ideal substrate for removing Pb^2+^ from aqueous solutions owing to its excellent hydrophilicity and ion exchange capacity. However, the limited adsorption selectivity and capacity of single carboxyl sites have restricted their application, and recent studies have widely adopted modification strategies such as biomass filler compounding, double-network copolymerization, and functional nanodoping. By introducing multiple active groups, including amino, sulfhydryl, and amide groups, these approaches have constructed efficient coordination and complexation networks, which not only significantly improve adsorption capacity and cycling stability but also endow the materials with smart properties, such as fluorescence sensing and photocatalytic conversion.

In the realm of sodium alginate-based composites and biomass utilization, researchers have significantly increased the diversity of adsorption sites by introducing exogenous components. Focusing on inorganic-organic composites, Zheng et al. [61] prepared Hydrogel-I by in situ embedding MAL powder into a sodium alginate matrix, as confirmed by FTIR, SEM, and XPS characterizations. Mechanistically, in addition to the inherent carboxyl groups of sodium alginate, the MAL powder provides abundant -OH, -NH, -NH_2_, and -CSS groups. These synergistically participate in the surface complexation and ion exchange of Pb^2+^. Furthermore, the material’s internal pore structure facilitates pore filling, with electrostatic attraction playing an auxiliary role. Exploring the introduction of polymer segments, Wang et al. [62] synthesized a sodium alginate-polyacrylamide (Alg-PAM) composite aerogel, characterized by FTIR, SEM, and XPS. This aerogel removed 99.2% of Pb^2+^ from wastewater, achieving a maximum capacity of 252.2 mg/g with negligible performance degradation after five adsorption–desorption cycles. The mechanism primarily relies on efficient chelation and ion exchange between multiple functional groups and Pb^2+^. Turning to low-cost biomass waste, Jacklien Emema Rose et al. [63] prepared a composite (SA-BPP) using banana peel powder and sodium alginate. Characterized via BET, FTIR, SEM-EDS, zeta potential, and TGA, the material exhibited a specific surface area of 28.308 m^2^/g. It achieved a 77% Pb^2+^ removal rate, maintaining 75% efficiency after five cycles. The endothermic adsorption process followed pseudo-first-order kinetics and the Freundlich isotherm, indicating multilayer adsorption where electrostatic interactions played a dominant role in cation capture. Similarly, the composite hydrogel microspheres prepared by Fagieh et al. [64] used sodium alginate and carrot waste carbon black, and were characterized by FESEM, XRD, and FTIR. Using sodium alginate and carrot waste carbon black, the Pb^2+^ adsorption process fits well with both the Langmuir isotherm (*R*^2^ = 0.977) and the pseudo-second-order kinetic model (*R*^2^ = 0.999), and the adsorption capacity of the hydrogel for Pb^2+^ reaches 57.82 mg/g. Adsorption of Pb^2+^ by Alg/carrot-Fe hydrogel is spontaneous and exothermic, and the prepared adsorbent can be reused for up to four cycles for Pb^2+^ adsorption in aqueous solution.

**Figure 4:**
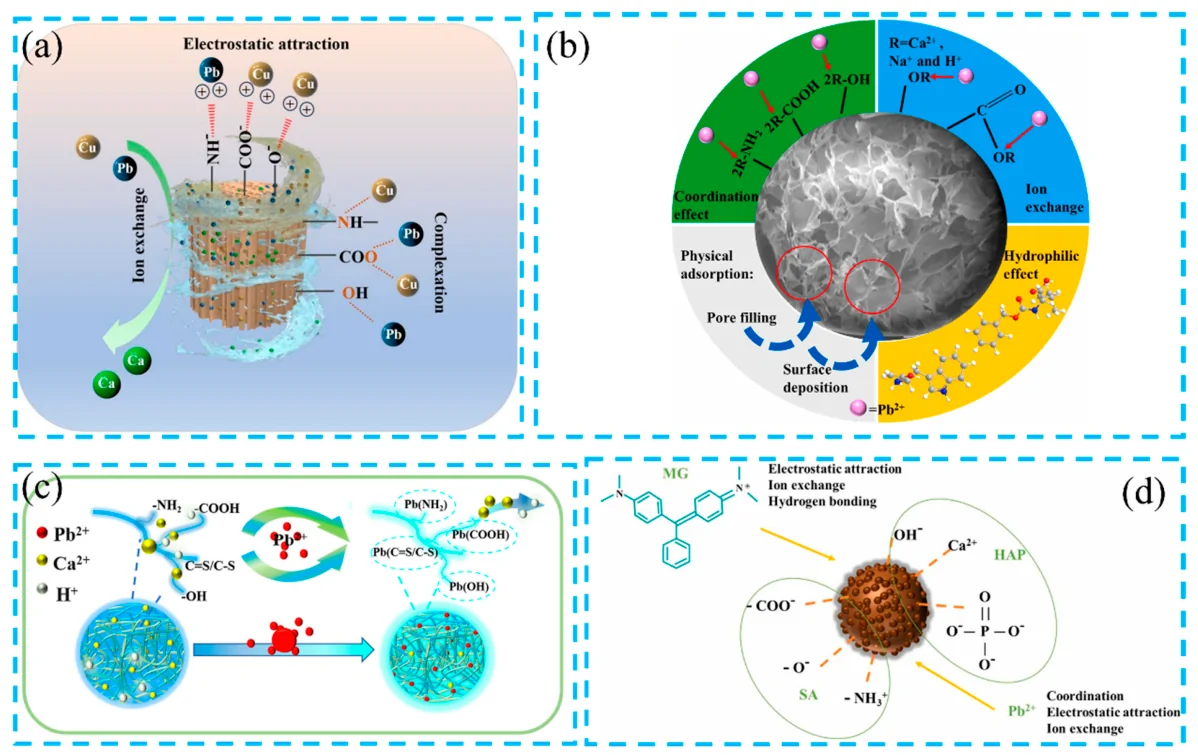
(**a**) Schematic diagram of the adsorption mechanism of STT. Reproduced with permission from reference [65]. Copyright 2025, Elsevier. (**b**) Adsorption mechanism of PCMC-SA-FC composite gel microspheres for Pb^2+^. Reproduced with permission from reference [66]. Copyright 2026, Elsevier. (**c**) Schematic diagram of the adsorption mechanism of SA/CS_2_/N-CDs gel beads. Reproduced with permission from reference [68]. Copyright 2025, Elsevier. (**d**) Adsorption mechanism of MHSB. Reproduced with permission from reference [69]. Copyright 2023, Elsevier.

In multi-component copolymerization strategies and double-network reinforcement, the introduction of macromolecular segments rich in active groups not only enhances mechanical properties but also alters the molecular-level adsorption mechanism. Regarding the incorporation of plant polyphenols, Li et al. [65] constructed a double-network aerogel (STT) using modified tannic acid (TA) and sodium alginate. Comprehensive characterizations (BET, FTIR, SEM, zeta potential, and XPS) revealed a maximum adsorption capacity of 422.80 mg/g, with efficiency remaining at 87.89% after 10 regeneration cycles. This process is predominantly governed by spontaneous monolayer chemisorption, consistent with the Langmuir model. Beyond conventional ion exchange, the phenolic hydroxyl groups of TA, along with modified amino and amide groups, undergo strong coordination with Pb^2+^, elevating both adsorption capacity and binding stability (as shown in Figure 4a). Exploring natural protein resources, Yang et al. [66] prepared composite hydrogel spheres (CMC-FC-SA) via a one-step physical blending method with Ca^2+^ cross-linking, verified by FTIR, SEM, zeta potential, and XPS. By innovatively using amino acid residues (e.g., amino and carboxyl groups) within the fish skin collagen (FC) as “ligand traps,” they achieved a high capacity of 586.56 mg/g. The behavior strictly conformed to the Langmuir isotherm and pseudo-second-order kinetics, indicating monolayer chemisorption. Mechanistically, ion exchange and ligand-metal interactions dominated, supplemented by physical adsorption (as shown in Figure 4b). Notably, specific amino acid side chains resisted interference from complex matrices via bidentate chelation. In contrast to the uniformly distributed adsorption sites described above, Noor et al. [67] developed magnetic graft copolymerized hydrogels (MHGs) and extensively characterized them using FTIR, FESEM, TEM, VSM, XRD, TGA, AFM, and BET. These MHGs exhibited a maximum capacity of 381.5 mg/g. Thermodynamic data indicated an endothermic process, while fitting to the Freundlich isotherm suggested the presence of energetically heterogeneous adsorption sites. Consequently, Pb^2+^ formed a multilayer on the MHG surface, predominantly via physical adsorption.

In cutting-edge research integrating intelligent detection and functional conversion, adsorption mechanisms have been further engineered to achieve specific advanced functions. Integrating adsorption and detection, Zhang et al. [68] prepared a fluorescent SA/CS_2_/N-CDs gel by combining amino-modified carbon dots with CS_2_-modified sodium alginate, as confirmed by FTIR, SEM, EDS, and XPS. This material achieved an ultra-high capacity of 607.08 mg/g. The core mechanism relies on the precise design of functional groups: thiol groups (-SH) introduced by CS_2_ modification possess a strong, specific affinity for heavy metals (as shown in Figure 4c). Alongside surface-enriched hydroxyl and carboxyl groups, these thiols firmly bind Pb^2+^ via multidentate chelation. Crucially, this complexation induces fluorescence quenching or a color change in the carbon dots, enabling simultaneous adsorption and visual detection. The data fit well with the Langmuir model, confirming uniform monolayer coverage. From the perspective of bionic structural design, Hua et al. [69] drew inspiration from dianthus pollen to prepare bionic hydrogel beads (MHSB) coated with magnetic hydroxyapatite. Characterized by FTIR, SEM, EDS, and XPS, this composite leveraged its unique microstructure to achieve an adsorption capacity of 475.50 mg/g. Further expanding on resource utilization, Liu et al. [70] designed SA/PAAS/PAC (SPP) microspheres and analyzed them via FTIR, SEM, EDS, XRD, and XPS. After adsorbing Pb^2+^ (capacity of 391.65 mg/g), PbS nanoparticles were formed via in situ vulcanization (as shown in Figure 4c). This innovative step converted the adsorption waste into a highly efficient photocatalyst for organic dye degradation, successfully realizing the environmental protection concept of “treating waste with waste.”

### 5.4. Chitosan-Based Hydrogels for Lead Ion Adsorption

Chitosan is a natural cationic polysaccharide derived from crustaceans. Owing to its highly reactive amino groups (-NH_2_) on its backbone and excellent biocompatibility, it has become an ideal matrix for capturing Pb^2+^ via strong coordination and ion exchange. Although it exhibits outstanding performance in low-concentration adsorption, current research mainly focuses on the optimization of modification strategies to overcome its limitations such as insufficient mechanical strength and difficulties in separation and recovery: on one hand, the introduction of magnetic particles and nanocellulose significantly enhances the material’s recovery efficiency and mechanical toughness; on the other hand, physical/chemical cross-linking is used to construct porous networks to improve adsorption kinetics. In addition, the specific functional design for electronic waste recycling and multi-ion co-existing systems also provides a new microscopic mechanistic perspective for solving complex water environmental pollution.

Regarding surface activity, separation, and recovery of adsorbents, researchers have endowed the materials with new properties through chemical modification. Samadzadeh Mamaghani et al. [40] developed a magnetic chitosan hydrogel (polyacrylamide nanocomposite) to address the challenges of trace lead ion removal and adsorbent recovery. This material exhibited an adsorption capacity of 31.74 mg/g for Pb^2+^, and the kinetic data fit the pseudo-second-order model, indicating that chemisorption was the rate-limiting step. Its advantage lies in its ability to achieve rapid magnetic separation, and it exhibits efficient, rapid adsorption kinetics in actual water samples.

Building on magnetic functionalization, the introduction of cellulose or inorganic nanomaterials has emerged as an effective strategy to further enhance the mechanical strength and adsorption site density of hydrogels. From the perspective of natural biomass fiber reinforcement, Yu et al. [71] prepared soy hull nanocellulose/sodium alginate/chitosan (SHNC/SA/CS) microspheres via ionic cross-linking. This material not only exhibited excellent toughness of 18.67 MPa but also demonstrated efficient adsorption of Pb^2+^ (116.62 mg/g) and Cu^2+^ (94.32 mg/g). The adsorption mechanism involves the synergistic effects of hydroxyl, carboxyl, and amino groups, including ion exchange, complexation, and electrostatic attraction. In similar cellulose modification studies, Xu et al. [72] also focused on cellulose modification, synthesizing a carboxymethyl chitosan/carboxylated nanocellulose (CYCS/CNC) hydrogel microsphere. This material substantially increased the surface negative charge density via carboxylation, creating efficient adsorption sites with strong electrostatic attraction for Pb^2+^. The adsorption capacity for Pb^2+^ reached 334.92 mg/g, and the adsorption process conformed to the Langmuir monolayer adsorption model and the pseudo-second-order kinetic model, indicating that the adsorption is limited by the saturated coverage of surface active sites, with chemical bonding as the dominant mechanism. Turning to the synergistic reinforcement strategy of inorganic nanofillers, Mirbagheri et al. [73] constructed a chitosan–sodium triphosphate–silica nanocomposite (CS-TPP-NSi). To confirm the successful integration of these components, the morphological and structural properties were systematically characterized using BET, FTIR, SEM, XRD, and XPS. During environmental remediation, the material exhibited rapid kinetics, removing 112.35 mg/g of lead. The characterization and performance data confirmed that the inclusion of nanosilica expands the specific surface area and facilitates the formation of synergistic adsorption centers between surface silanol groups (Si–OH) and chitosan amino groups, with monolayer adsorption functioning as the primary mechanism.

After the composite components are determined, different cross-linking methods directly determine the pore structure and chemical stability of the adsorbent (Table 3). Starting from the macropore construction strategy of physical cross-linking, Nankawa et al. [74] prepared spongy chitosan hydrogels with a macroporous structure (approximately 100 μm) via the freeze–thaw physical cross-linking method in an alkaline solution. This method uses ice crystal growth as a pore-forming template to create an interconnected three-dimensional pore network, thereby significantly reducing mass transfer resistance. The material not only exhibits an extremely high adsorption capacity for Pb^2+^ (192.3 mg/g), but also offers a unique visual monitoring function: its color changes after Cu^2+^ adsorption, and it can be regenerated by simple compression and rinsing. Unlike physical cross-linking, chemical cross-linking strategies primarily focus on improving structural stability. Wang et al. [75] synthesized three-dimensional macroporous hydrogel beads by blending chitosan with acrylic acid using formaldehyde as a cross-linking agent. The successful formation of the porous network and the chemical functionalization were verified via FTIR, SEM, and XPS. Formaldehyde links the polymer chains via Schiff base reactions, enhancing acid and alkali resistance while preserving porosity. Adsorption studies demonstrated that the amino groups are the primary active sites. The capture process followed the Freundlich model and pseudo-second-order kinetics, with chemical adsorption as the dominant mechanism. In a related comparative study, Ayach et al. [76] compared the properties of raw chitosan powder and its chemically cross-linked hydrogel. The structural transitions were comprehensively evaluated using BET, FTIR, SEM, XRD, and NMR. They found that the extracted chitosan hydrogel achieved a lead removal rate of 95%. Mechanism analysis indicated that the powder form mainly relies on physical adsorption via van der Waals forces, whereas covalent bonds introduced after cross-linking increase the proportion of chemical adsorption.

For complex contamination scenarios in practical applications, particularly when dealing with complex systems such as electronic waste (E-waste) and co-contaminated water bodies, the design specificity of adsorbents is of paramount importance (Table 3). In the field of resource recycling of electronic waste, Sivakumar et al. [77] prepared surface-modified hydrogels by blending polyvinyl alcohol (PVA) and chitosan with conductive polymers (polyaniline, PANI, and polypyrrole, PPy). The physicochemical properties and surface topology of the functionalized hydrogels were analyzed using BET, FTIR, and SEM. These hydrogels were specifically designed for the recovery of Pb^2+^ and Cd^2+^ from waste-printed circuit board solutions. The π-electron conjugated systems of the conductive polymers not only provide additional coordination sites but also enhance the selective capture of polyvalent metal ions through redox reactions. This material exhibited excellent thermal stability and achieved metal ion recovery rates exceeding 90%. Notably, the adsorption behavior of this material in multi-metal coexistence systems was more consistent with the characteristics of multi-layer adsorption (as described by the Freundlich model).

Regarding co-existing anion interference in agricultural non-point source pollution, He et al. [78] synthesized a chitosan-chlorite bio-nanocomposite (CGB). The material’s crystallographic and morphological characteristics were comprehensively examined through Mössbauer spectroscopy, XRD, and SEM. This CGB demonstrated a high adsorption capacity for Pb^2+^ (66.76 mg/g). Through research, it was found that in co-contaminated systems containing phosphate, phosphate can eliminate the electrostatic shielding effect and act as an “ion bridge,” triggering spatially self-stratified synergistic adsorption of Pb^2+^ and Cu^2+^ (with copper enriched at the surface and lead at the core). This behavior led to a significant increase in total adsorption capacity in ternary systems compared to single-metal systems. This discovery challenges the traditional perception that co-existing ions solely produce competitive inhibition, thereby providing new microscopic mechanistic evidence for addressing agricultural non-point source pollution.

### 5.5. Starch-Based Hydrogels for Lead Ion Adsorption

Starch is a widely available natural non-ionic polysaccharide with a molecular chain rich in hydroxyl groups, exhibiting excellent hydrophilicity and potential for chemical modification. Starch-based hydrogels constructed through physical entanglement or chemical cross-linking can significantly enhance their adsorption capacity for heavy metal ions upon introduction of active functional groups such as carboxyl, phosphate, or amino groups. Furthermore, composite systems incorporating inorganic minerals, nanomaterials, or conductive polymers can enhance mechanical strength and recyclability, positioning them as highly promising green adsorbents for environmental remediation.

To endow starch with stronger ion-capture capabilities, researchers commonly employ graft copolymerization to introduce highly active functional groups. Aniagor et al. [79] synthesized a “c-hydrogel” with superabsorbent properties by hydrolyzing polyacrylonitrile (PAN)-grafted starch. The structural transformations during synthesis were confirmed using FTIR and SEM. By converting nitrile groups into amide and carboxyl groups, this material achieved rapid Pb^2+^ adsorption (reaching equilibrium within 40 min) with a maximum adsorption capacity of 264.37 mg/g. The adsorption process followed the Langmuir model and pseudo-first-order kinetics. Building on this, further studies have focused on improving adsorption performance by introducing inorganic fillers or optimizing functional-group conversion strategies. Hou et al. [80] developed a starch–tobermorite composite hydrogel (LR-TOB/AA). The morphology and elemental composition of the composite were systematically characterized using FTIR, SEM, EDS, XRD, and XPS. The introduction of tobermorite significantly enhanced the thermal stability and structural strength of the hydrogel. Performance tests revealed that the adsorption capacity for Pb^2+^ reached 900.25 mg/g, and the adsorption efficiency remained above 90% after five reuse cycles. The adsorption mechanism primarily involves electrostatic attraction and coordination complexation between Pb^2+^ and the carboxyl, hydroxyl, and amino groups. Similarly, Lin et al. [81] developed a corn starch/acrylic acid/isoamyl acetate (CS/AA/IA) ion-exchange hydrogel, wherein carboxyl groups were converted into highly active –COONa groups via alkali washing. The structural and surface properties of the hydrogel were verified via FTIR, SEM, XRD, and XPS. The prepared material exhibited exceptional adsorption capacities of 869.56 mg/g for Pb^2+^ and 699.31 mg/g for Cu^2+^, maintaining a 95% adsorption efficiency after five cycles. The primary mechanism was confirmed to be a highly efficient ion-exchange process.

Beyond the pursuit of high adsorption capacity, endowing hydrogels with specialized functions—such as sensing capabilities or enhanced stability—has become a research hotspot. The thermal stability and adsorption performance of materials can be simultaneously improved by introducing cross-linkers with specific structures. Almohammed et al. [82] prepared a starch acrylate hydrogel (StA-OTCSS) using an eight-arm (3-mercaptopropyl) trisiloxane as the cross-linker. The chemical structure and morphology of the hydrogel were comprehensively analyzed using ^1^H NMR, FTIR, SEM, and EDX. The introduction of siloxane significantly improved the material’s thermal stability and the accessibility of surface-active sites, enabling adsorption capacities of 381.30 mg/g and 366.80 mg/g for Pb^2+^ and Cd^2+^, respectively. The adsorption process was endothermic and spontaneous, conforming to the Langmuir monolayer adsorption model. Furthermore, integrating fluorescent materials and nanoreinforcements into starch matrices can enable the dual function of adsorption and detection. Zhang et al. [83] innovatively introduced carbon dots (CDs) and cellulose nanofibers (CNs) into a starch matrix to synthesize a fluorescent hydrogel (FSH). The successful integration of these components was confirmed via FTIR, SEM, XRD, and XPS. This material not only exhibited an adsorption capacity of 265.9 mg/g for Pb^2+^ but also enabled highly sensitive detection of Pb^2+^ via fluorescence quenching induced by photoinduced electron transfer (PET), achieving a detection limit of 0.06 μg/L. The mechanism was driven by a combination of ion exchange, electrostatic interactions, and complexation, successfully enabling simultaneous adsorption and detection.

To address the challenges associated with modifying starch from specific plant sources and with adsorbent recovery, scholars have proposed targeted solutions. For natural starch, issues such as easy swelling, low mechanical strength, and insufficient stability can be effectively mitigated through cross-linking and chemical modification. Akinterinwa et al. [84] selected native adzuki bean starch to prepare a modified derivative (SCCS) through sodium trimetaphosphate cross-linking and carboxymethylation. The structural modifications were characterized using FTIR and SEM-EDX. This modification significantly improved the material’s stability in aqueous solutions. The SCCS not only exhibited an extremely fast adsorption rate (reaching equilibrium within 20 min) but also demonstrated excellent removal of low-concentration (5 ppm) Pb^2+^, with the adsorption efficiency remaining high (88.78%) after five reuse cycles. The adsorption process followed pseudo-second-order kinetics and was mainly driven by coordination complexation and ion exchange between the metal ions and the –OH, –COO^−^, and P–O groups. In practical applications, the separation and recovery of adsorbents remain key bottlenecks limiting their widespread adoption; magnetic functionalization provides an effective solution to this issue. Perez et al. [85] designed a magnetic nanoadsorbent consisting of a manganese ferrite (MnFe_2_O_4_) nanoparticle core with an ultrathin carboxymethyl starch coating (approximately 4% by weight). The core–shell structure and surface properties were evaluated using TEM, AFM, FTIR, and zeta potential measurements. Although the organic layer is extremely thin, it provides abundant heterogeneous surface-active sites, achieving efficient Pb^2+^ removal (~100%) with an adsorption capacity of 34–46 mg/g, while retaining a high saturation magnetization of 65 emu/g. These properties enable rapid adsorbent separation and regeneration using an external magnetic field, demonstrating the high efficiency of surface functionalization in nanoadsorbent design.

### 5.6. Composite Hydrogels for Lead Ion Adsorption

Composite hydrogels are constructed by blending two or more biomass materials (such as cellulose, chitosan, lignin, and sodium alginate) via physical mixing or chemical cross-linking. This composite strategy integrates the advantageous properties of each component, achieves synergistic adsorption by introducing diverse functional groups and increasing the specific surface area, significantly enhances both the capture capacity for Pb^2+^ and the material’s mechanical properties, and has become a research hotspot in the field of heavy-metal removal from water bodies.

**Figure 5:**
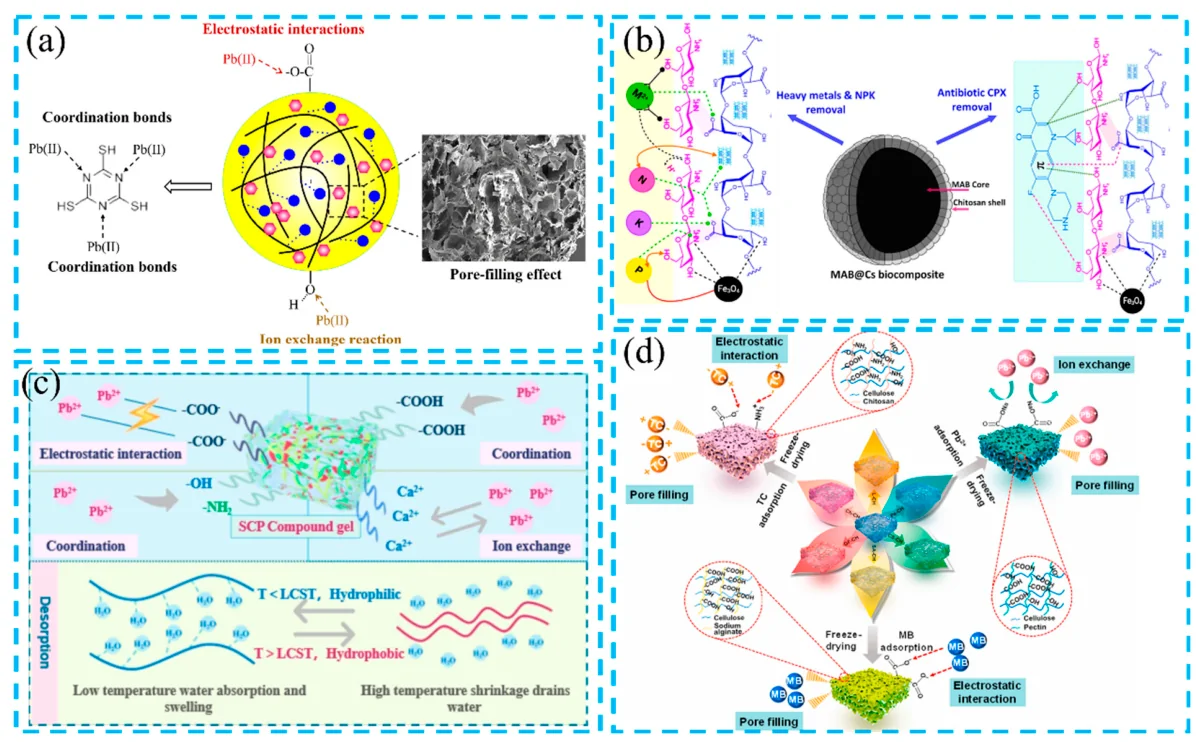
(**a**) Schematic diagram of the adsorption mechanism of Pb^2+^ by Hydrogel-II. Reproduced with permission from reference [86]. Copyright 2024, RSC Advances. (**b**) Adsorption mechanism of organic and inorganic pollutants by mAB@Cs. Reproduced with permission from reference [90]. Copyright 2024, Elsevier. (**c**) Schematic diagram of the adsorption mechanism of Pb^2+^ by SCP composite hydrogel. Reproduced with permission from reference [92]. Copyright 2025, Elsevier. (**d**) Adsorption mechanism of MB, Pb^2+^ , and TC by hydrogel. Reproduced with permission from reference [93]. Copyright 2022, Elsevier.

Combining industrial by-product lignin with sodium alginate can not only reduce production costs but also improve the adsorbent’s applicability in dynamic treatment. Wang et al. [86] blended alkali lignin (AL), trithiocyanuric acid trisodium salt (TMT), and sodium alginate (SA) to prepare a composite hydrogel bead, which was applied in a fixed-bed column to treat wastewater containing Pb^2+^. Structural and morphological characterization was performed using FTIR and SEM. The study showed that the breakthrough curve of the adsorbent was significantly affected by flow rate and bed height, and that it still exhibited good regeneration ability after three adsorption–desorption cycles, demonstrating its potential for practical continuous-flow treatment (as shown in Figure 5a). To further optimize the dynamic adsorption performance of fixed beds, researchers have begun to focus on the effect of influent flow mode on adsorption efficiency. On this basis, Zheng et al. [87] developed “Hydrogel-I,” composed of modified alkali lignin (MAL) and sodium alginate. The composite’s chemical and surface properties were verified via FTIR and SEM. Investigation of its dynamic adsorption behavior in a fixed bed showed that the bottom-up influent mode allowed more effective contact between the adsorbent and pollutants, achieving a saturated adsorption capacity of 678 mg/L—remarkably higher than that observed in the top-down mode. The abundant functional groups, including –NH/–NH_2_, C=S, and C–S, served as the main active adsorption sites. The material exhibited excellent stability and reusability over three cycles.

Beyond fixed-bed systems, controlling the composite morphology—such as membrane or core–shell structures—of chitosan and sodium alginate can significantly enhance mechanical strength and physicochemical properties, thereby expanding their application potential. Moreno-Rivas et al. [88] fabricated sodium alginate/chitosan interpenetrating network gel membranes using solvent casting. Chemical composition and morphology were characterized by FTIR, SEM, thermogravimetric analysis (TGA), and differential scanning calorimetry (DSC). The membranes demonstrated pH-responsive swelling behavior and achieved a 98% Pb^2+^ removal efficiency at pH 6.5 via electrostatic interactions and ion-exchange mechanisms. Adsorption kinetics and isotherms for Cd^2+^ and Pb^2+^ followed the Langmuir model and pseudo-second-order kinetics. High desorption efficiency was maintained after five reuse cycles. In comparison, core–shell structures enable more effective integration of multiple functional components and synergistic improvements in mechanical properties and adsorption performance. Dong et al. [89] prepared double-enhanced core–shell particles (Ca-SA-WSC@WSC/PVA) by combining water-soluble chitosan (WSC), polyvinyl alcohol (PVA), and sodium alginate matrices via freezing/thawing cycles and ionic crosslinking. FTIR and SEM-EDS analyses confirmed the composite structure. This core-shell material exhibited significantly improved storage modulus and thermal stability. It simultaneously adsorbed CO_2_ and Pb^2+^ (39.28 mg/g) and Cu^2+^, demonstrating excellent multifunctional potential for environmental remediation.

Although these materials exhibit promising adsorption performance, challenges of difficult adsorbent separation and insufficient detection sensitivity for trace pollutants persist in practical applications. To overcome these limitations, introducing functional nanoparticles has become an effective strategy. Chkirida et al. [90] designed a biocomposite with a magnetic core–shell structure (mAB@Cs) consisting of an alginate-bentonite core and chitosan-aspartic acid shell, with Fe_3_O_4_ nanoparticles generated in situ. The composite was characterized by FTIR, SEM-EDX, and XRD. Based on a monolayer chemisorption mechanism, the material achieved removal efficiencies ranging from 74.89% to 99.86% for seven heavy metals, including Pb^2+^. Additionally, it effectively removed organic pollutants and pathogenic bacteria. Its magnetic property facilitated easy separation from aqueous solutions (as shown in Figure 5b). While magnetic separation addresses macro-scale adsorption recovery, the development of efficient solid-phase extraction (SPE) materials is essential for trace-level heavy metal analysis. Siswanta et al. [91] synthesized an alginate-chitosan-carbon (CAC) composite adsorbent for SPE enrichment of trace Pb^2+^ in water. The material was characterized structurally and functionally via FTIR and SEM. Under optimized conditions (pH 6), the recovery rate of Pb^2+^ reached 93–96%. Coupled with flame atomic absorption spectrometry (FAAS), it achieved a detection limit of 0.06 μg/L, demonstrating its efficacy as a pretreatment method for ultra-trace heavy metal detection in wastewater.

Beyond functional modification, imparting environmental responsiveness to hydrogels and utilizing agricultural waste for modification represent innovative paths for controllable adsorption and sustainable development. Wang et al. [92] introduced the thermosensitive polymer poly (N-isopropylacrylamide) (PNIPAM) into sodium alginate (SA) and carboxymethyl cellulose (CMC) matrices to prepare a thermoresponsive composite hydrogel with an interpenetrating network structure. Material characterization included FTIR, SEM, and swelling tests. By regulating surface hydrophilicity/hydrophobicity via temperature variation, the hydrogel exposes hydrophilic groups at low temperatures to capture Pb^2+^ (with an adsorption capacity of up to 311 mg/g). Upon heating, conformational changes release metal ions, enabling intelligent adsorption-regeneration control (as shown in Figure 5c). In addition to enhancing performance by introducing intelligent, responsive units, using low-cost agricultural waste as raw materials is another important direction for the sustainable development of hydrogel adsorbents. Lei et al. [93] used biomass resources by cross-linking corn straw cellulose with heteropolysaccharides, such as xylan and chitosan, to fabricate modified composite hydrogels. The structural features were confirmed by FTIR, SEM, and EDX. The composites exhibited significantly increased specific surface area. Through mechanisms including electrostatic interactions, pore filling, and ion exchange, their Pb^2+^ adsorption capacity increased from 1.73 mg/g to 30.03 mg/g, providing a novel approach to valorizing agricultural waste in water treatment (as shown in Figure 5d).

In practical wastewater treatment, the adsorption performance of composite hydrogels toward Pb^2+^ is influenced by the presence of common competing ions. Divalent cations such as Ca^2+^, Mg^2+^, Cu^2+^, Cd^2+^, and Zn^2+^ may compete for active adsorption sites via electrostatic interactions or complexation, potentially reducing Pb^2+^ uptake. Considering these competitive adsorption effects is essential for more accurately evaluating the selectivity and practical applicability of biomass-based composite hydrogels for Pb^2+^ removal in complex aqueous matrices.

## 6. Conclusions and Outlook

Biomass-based hydrogels, as a class of green and sustainable adsorbents, exhibit immense potential for lead ion removal. Cellulose hydrogels have significantly enhanced their adsorption capacity through modification strategies such as carboxylation and amination. Lignin hydrogels achieve efficient lead ion capture by leveraging their abundant phenolic hydroxyl and carboxyl active sites. Sodium alginate hydrogels demonstrate excellent selectivity for Pb^2+^ via ion exchange between carboxyl groups and Pb^2+^. Chitosan hydrogels, meanwhile, exhibit favorable adsorption performance in weakly acidic environments through the synergistic action of amino and hydroxyl groups. Additionally, starch-based hydrogels offer a highly modifiable backbone, enabling the grafting of diverse functional groups to effectively trap heavy metal ions. Composite hydrogels further enhance the mechanical strength and adsorption performance of materials through multi-component synergistic effects. Notably, the application of technologies such as fixed-bed dynamic adsorption and core–shell structure design has provided feasible pathways for practical wastewater treatment. Studies on adsorption mechanisms indicate that lead ion removal by biomass-based hydrogels primarily involves the synergistic action of multiple mechanisms, including electrostatic interaction, ion exchange, complexation, and physical adsorption. Among these, sodium alginate primarily relies on ion exchange, chitosan primarily on chelation, and lignin primarily on hydrogen bonding. The adsorption process generally conforms to the Langmuir isothermal adsorption model and the pseudo-second-order kinetic model, indicating that adsorption is primarily monolayer chemical adsorption. Environmental factors such as pH, temperature, and initial concentration significantly influence adsorption performance. Optimizing these parameters can effectively enhance the practical application efficacy of the materials. To better connect laboratory findings with practical applications, future research on hydrogel adsorbents should focus on their performance and durability under real wastewater conditions. This includes improving mechanical stability to withstand continuous flow and shear forces typical in industrial treatments, enhancing regeneration methods to maintain adsorption capacity over multiple cycles without damaging the hydrogel structure, and developing safe, sustainable disposal strategies for exhausted Pb-loaded hydrogels to prevent secondary pollution. Addressing these challenges is essential for advancing hydrogel technologies toward real-world use.

Future research on biomass-based hydrogels for lead adsorption should focus on integrating material innovation with performance optimization. The development of multi-functional composite materials is an important research direction. By introducing functional components such as magnetic nanoparticles, conductive polymers, carbon nanotubes, or graphene, hydrogels can be endowed with multiple functions, including magnetic separation, sensing, detection, and photocatalytic degradation, thereby enabling integrated “adsorption-detection-degradation” treatment. The design of smart responsive hydrogels also deserves attention. The development of pH-, temperature-, or photo-responsive hydrogels can enable controllable switching between adsorption and desorption, improve regeneration efficiency, and reduce energy consumption, thereby reducing chemical reagent consumption. The application of “ion-imprinting technology (IIT)” enables hydrogels to form adsorption structures with specific recognition sites, thereby significantly improving the selective adsorption capacity for Pb^2+^ and addressing competitive adsorption in environments containing multiple co-existing ions. The resource utilization of agricultural waste is of great economic and environmental significance. It is necessary to fully exploit low-cost biomass resources, such as straw, nut shells, and rice husks, to prepare high-performance adsorbents through appropriate chemical or physical modification, and to realize the high-value utilization of these wastes. In-depth investigation of the adsorption mechanism requires advanced characterization techniques such as in situ XPS, EXAFS, and molecular dynamics simulations to reveal the microscopic interactions between metal ions and functional groups during the adsorption process and to establish structure-performance relationship models, thereby providing theoretical guidance for the rational design of high-efficiency adsorbents. Optimizing continuous-flow treatment processes is crucial for engineering applications. It is necessary to deepen the research on dynamic adsorption processes, such as fixed beds and membrane reactors, systematically evaluate the influence of operating parameters, such as bed height, flow rate, and feed concentration, on adsorption performance, and assess the long-term stability and economic feasibility of biomass hydrogels in actual wastewater treatment, to promote their transformation from laboratory research to industrial application. Finally, to genuinely achieve environmental sustainability and prevent secondary pollution, future studies must prioritize the end-of-life management of these adsorbents. Comprehensive Life Cycle Assessments (LCAs) should be integrated to trace the ultimate fate of both materials and heavy metals. This includes developing safe recovery strategies for the highly concentrated Pb^2+^ eluent generated during desorption (e.g., via chemical precipitation or electrodeposition), as well as establishing secure disposal methods for the exhausted hydrogels (e.g., incineration for energy recovery or solidification/stabilization), rather than relying solely on natural biodegradation.

## Figures and Tables

**Table 2:** Summary of Lignin biomass-based adsorptive hydrogels/aerogels/composite gels for Pb^2+^.

Material Type	Description	Max Adsorption Capacity (mg/g)	Optimal pH	Isotherm Model	Kinetic Model	Adsorption Mechanism	Reference
AHL-g-PAA Hydrogel	Acid hydrolyzed lignin grafted on polyacrylic acid network	235	/	Langmuir	Pseudo-second-order	Monolayer chemisorption	[52]
Lignocellulose Gel (LCG)	Hydrogels with different lignin content via the dissolution method	86.1	/	/	/	Chemisorption and multi-step diffusion	[53]
Lignocellulose Microbeads	Microbeads with residual lignin promoting pores & OH groups	~34 (0.161 mmol/g)	/	/	/	Pore formation enhanced adsorption	[54]
rGO@LNP Composite Hydrogel	Reduced graphene oxide + lignin nanoparticles from walnut shell	/	5	/	/	Chelation + Lewis acid-base interactions	[55]
LS-GH Hydrogel	Lignosulfonate-modified graphene hydrogel	1210 (static), 1308 (dynamic)	/	/	/	3D porous, oxygen functional groups	[56]
LBPAA/OrgMMT Nanocomposite	Lignin-grafted copolymer + organic montmorillonite	/	/	Freundlich	Pseudo-second-order	Chemical complexation of Pb–O bonds	[57]
BLPA-MA Hydrogel	Bentonite/sodium lignosulfonate copolymer hydrogel	/	/	Langmuir	Pseudo-second-order	Monolayer chelation + ion exchange	[58]
Ls-AA-AM-MBA Copolymer	Graft copolymer of lignosulfonate, acrylic acid, and acrylamide	111.94	/	Langmuir	Pseudo-second-order	Pore size-dependent chemisorption	[59]
Fe_3_O_4_@Lignin Hydrogel	Magnetic Fe_3_O_4_ combined with lignin for soil remediation	/	/	/	/	Multiple mechanisms: adsorption, ion exchange, precipitation	[60]

Notes: / indicates data not specified or unavailable in original reports. Adsorption mechanisms often involve chemisorption/chelation due to lignin’s phenolic and carboxyl groups, combined with structural factors such as porosity and composite synergy.

**Table 3:** Summary of Chitosan biomass-based adsorptive hydrogels/aerogels/composite gels for Pb^2+^.

Material	Modification/Structure Strategy	Max Adsorption Capacity	Isotherm Model	Kinetic Model	Main Mechanism	Reference
Magnetic chitosan hydrogel (polyacrylamide nanocomposite)	Magnetic functionalization for fast separation + PAAm composite	31.74 mg/g	/	Pseudo-second-order	Chemisorption as rate-limiting step; fast magnetic recovery	[40]
SHNC/SA/CS microspheres (soy hull nanocellulose/sodium alginate/chitosan)	Ionic cross-linking; natural biomass fiber reinforcement; tougher microspheres	Pb^2+^: 116.62 mg/g; Cu^2+^: 94.32 mg/g	/	/	Synergy of –OH, –COOH, –NH_2_: ion exchange, complexation, electrostatic attraction	[71]
CYCS/CNC hydrogel microsphere (carboxymethyl chitosan/carboxylated nanocellulose)	Carboxylation to increase negative charge + cellulose nanofiller	334.92 mg/g	Langmuir	Pseudo-second-order	Limited by saturated coverage of active sites, chemical bonding is dominant.	[72]
CS-TPP-NSi nanocomposite (chitosan–triphosphate–nanosilica)	Inorganic nanofiller reinforcement (nanosilica) + inorganic bridging (TPP)	112.35 mg/g	Monolayer	/	Synergistic centers: Si–OH + chitosan amino; monolayer adsorption primary	[73]
Spongy macroporous chitosan hydrogel (freeze–thaw physical cross-linking)	Physical cross-linking + macropore templating via ice crystal growth	192.3 mg/g	/	/	Reduced mass-transfer resistance; physical adsorption/recovery regeneration described; visible color change after Cu^2+^ adsorption	[74]
3D macroporous chitosan/acrylic acid hydrogel beads (formaldehyde cross-linking)	Chemical cross-linking using formaldehyde (Schiff base)	/	Freundlich	Pseudo-second-order	Schiff base improves stability; amino groups are the main active sites; strong chemical adsorption is controlled.	[75]
Cross-linked chitosan hydrogel vs. raw chitosan powder (comparative)	Compare powder (physical) vs. covalently cross-linked hydrogel	95% removal rate	/	/	Powder: physical adsorption (van der Waals). Cross-linking: more covalent/chemical adsorption	[76]
PVA/chitosan + conductive polymer hydrogels (PANI, PPy; PVA matrix)	E-waste recycling-oriented selective capture; conductive polymers for extra coordination + redox	>90% recovery rate	Freundlich	/	Conductive polymers provide coordination sites and redox-driven selective capture; multi-layer behavior dominates	[77]
Chitosan-chlorite bio-nanocomposite (CGB)	Co-contaminant ion-bridge concept; system-level synergy with phosphate	66.76 mg/g	/	/	In phosphate-containing systems, phosphate removes electrostatic shielding and acts as an “ion bridge,” enabling spatial self-stratification (Cu at the surface, Pb at the core).	[78]

Note: PAAm Polyacrylamide; SHNC Soy hull nanocellulose; SA Sodium alginate; CS Chitosan; CYCS Carboxymethyl chitosan; CNC Carboxylated nanocellulose; TPP Sodium triphosphate; NSi Nanosilica; PVA Polyvinyl alcohol; PANI Polyaniline; PPy Polypyrrole; CGB Chitosan-chlorite bio-nanocomposite.

## Data Availability

All the data are provided in this manuscript.
